# A systematic study of independently-tuned room-specific PBS beam model in a beam-matched multiroom proton therapy system

**DOI:** 10.1186/s13014-021-01932-0

**Published:** 2021-10-29

**Authors:** Yu-Hua Huang, Chunfeng Fang, Tao Yang, Lin Cao, Gaolong Zhang, Baolin Qu, Yihang Zhang, Zishen Wang, Shouping Xu

**Affiliations:** 1 Department of Radiation Oncology, The First Medical Center of PLA General Hospital, Beijing, 100853 China; 2 Department of Health Technology and Informatics, The Hong Kong Polytechnic University, Kowloon, Hong Kong; 3 Department of Radiation Oncology, Hebei Yizhou Cancer Hospital, Zhuozhou, 072750 China; 4 School of Physics, Beihang University, Beijing, 100191 China

**Keywords:** Beam-matching, Beam analysis, Proton therapy, Pencil beam scanning, Monte Carlo

## Abstract

**Background:**

In the existing application of beam-matched multiroom proton therapy system, the model based on the commissioning data from the leading treatment room was used as the shared model. The purpose of this study is to investigate the ability of independently-tuned room-specific beam models of beam-matched gantries to reproduce the agreement between gantries’ performance when considering the errors introduced by the modeling process.

**Methods:**

Raw measurements of two gantries’ dosimetric characteristics were quantitatively compared to ensure their agreement after initially beam-matched. Two gantries’ beam model parameters, as well as the model-based computed dosimetric characteristics, were analyzed to study the introduced errors and gantries’ post-modeling consistency. We forced two gantries to share the same beam model. The model-sharing patient-specific quality assurance (QA) tasks were retrospectively performed with 36 cancer patients to study the clinical impact of beam model discrepancies.

**Results:**

Intra-gantry comparisons demonstrate that the modeling process introduced the errors to a certain extent indeed, which made the model-based reproduced results deviate from the raw measurements. Among them, the deviation introduced to the IDD curves was generally larger than that to the beam spots during modeling. Cross-gantry comparisons show that, from the beam model perspective, the introduced deviations deteriorated the high agreement of the dosimetric characteristics originally shown between two beam-matched gantries, but the cross-gantry discrepancy was still within the clinically acceptable tolerance. In model-sharing patient-specific QA, for the particular gantry, the beam model usage for intensity-modulated proton therapy (IMPT) QA plan generation had no significant effect on the actual delivering performance. All reached a high level of 95.0% passing rate with a 3 mm/3% criterion.

**Conclusions:**

It was preliminary recognized that among beam-matched gantries, the independently-tuned room-specific beam model from any gantry is reasonable to be chosen as the shared beam model without affecting the treatment efficacy.

## Introduction

The number of proton therapy equipment in the world is still far smaller than that of conventional radiotherapy equipment nowadays, especially in developing countries [1]. For multiroom proton treatment systems, even though the proton beams delivered in each treatment room are generated from the same cyclotron/synchrotron and share the same set of energy adjustment, degrader, energy selection system (ESS) and part of the beamline, their beam dosimetric characteristics cannot be guaranteed to be exactly identic due to the differences in the beam transporting distance and the general deviations that may occur in their gantries and nozzles. Therefore, in theory, a specific machine model, which is measured, built and tuned in the treatment planning system (TPS) separately for each room, could accurately describe the corresponding treatment room’s condition to the most extent.

The beam-matching concept was first proposed for conventional photon and electron radiotherapy. Vendors make the dosimetric and electromechanical properties similarities among two or more linear accelerators (Linacs) reach specific criteria after fine-tunes. Patients could be arranged to use any Linac for continuous treatment without replanning or recalculating treatment plans, which significantly improves the treatment flexibility and efficiency [2]. Moreover, for beam-matched Linacs, only one shared machine model needs to be built. In recent years, vendors also began to provide (quasi-) beam-matching options for multiroom proton therapy systems based on their criteria, which is generally generous and various among vendors. Due to the lack of a consensus beam-matching guideline, its relevant studies in proton therapy were rarely reported [3, 4]. Recent research by Rana et al. [4] carried out in IBA ProteusPLUS multiroom proton therapy system (IBA, Louvain-la-Neuve, Belgium) reported that several pencil beam scanning (PBS) dosimetric characteristics of three beam-matched gantries were feasible to match within clinically acceptable tolerances.

For the practical clinical application of beam-matching in proton therapy, in addition to achieving high consistency in beam dosimetric characteristics among rooms, an appropriate model which could theoretically represent all gantries in TPS is also necessary. The model for a particular PBS delivery system typically consists of machine-related parameters (hardware geometrical and material properties, etc.) and beam-related parameters (beam effective energy spectra, beam optics, beam meterset calibrations, virtual source axis distances, etc.) [5]. Beam modeling is a parameterization process to fit those raw measurements of actual beam dosimetric characteristics in terms of several beam model parameters and make the computed beam dosimetric characteristics (as dosimetric representations of modeling results) reproduced by the dose calculation algorithm close to the respective raw measurements, through fine-tuning the beam model parameters. As an abstraction and approximation of the actual physical process, it is necessary to make a trade-off in model complexity, time-labor costs and accuracy in most cases, which makes the built model usually unable to reproduce the actual beam characteristics perfectly.

The existing clinical practice was, the TPS model based on the commissioning data from the leading treatment room would be used as the shared model for other matched treatment rooms without further adjustment [4]. A thing not taken into account is whether the shared model from one of those beam-matched gantries still can accurately describe all gantries’ conditions when considering the errors introduced by the modeling process. Unlike the study mentioned above, the quasi-beam-matched gantries in our hospital’s multiroom system initially used a room-specific beam model for each room for planning and treatment. Beam models of two rotating gantry treatment rooms (TR2 and TR4) were tuned independently without reference to each other, which allows us to investigate under the premise that these gantries have been beam-matched by the vendor, the ability of independently-tuned room-specific beam models to reproduce the agreement between gantries’ performance. The study covers the following aspects. (a) Raw measurements of two gantries’ dosimetric characteristics were quantitatively compared to ensure their agreement after initially beam-matched. (b) Two gantries’ beam model parameters and the computed dosimetric characteristics (as the representations of beam models) were analyzed to study the introduced errors and gantries’ consistency after the modeling process. (c) We forced two gantries to share the same beam model. Patient-specific quality assurance (QA) tasks were performed to study the clinical impact of beam model deviations. Our results will provide a general reference to the beam-matching application from the model perspective.

## Materials and methods

### General study design

Our hospital (Yizhou Cancer Hospital, Hebei Province, P. R. China) has five proton treatment rooms (TR1 to TR5), and is equipped with ProteusPLUS cyclotron-based multiroom proton therapy system. The TR1 is a fix-beam treatment room, while the TR2–TR4 are rotating gantry treatment rooms using the PBS technique with the IBA PBS dedicated nozzles (DNs). And the TR5 is a rotating gantry treatment room using both PBS and double scattering (DS) techniques with IBA universal nozzle (UN). In this proton system, a C230 cyclotron was used to produce continuous high-energy proton beams with constant energy [6], and the proton energy was adjusted to the required range by degrader and ESS. The minimum and the maximum energies of protons are 69.8 MeV (range: 4.0 g/cm^2^) and 226.09 MeV (range: 32.0 g/cm^2^), respectively. Our study was carried out in two of three rotating gantry treatment rooms with DNs, TR2 and TR4 (with GTR2 and GTR4), which have completed the acceptance tests and commissioning before [7, 8]. A detailed room-specific PBS machine model was built for each gantry in RayPhysics module to be used in treatment planning in RayStation TPS (Version 7.10, RaySearch Laboratories, Stockholm, Sweden). Among the beam-related parameters included in the model, only integrated depth dose (IDD) distribution (the dosimetric representation of beam effective energy spectra), and spot profiles (the dosimetric representation of beam optics) were contained in this study. There are two reasons for this consideration. First, the virtual source axis distances ($SAD_{vir}$) are constant values for a particular gantry over the therapeutic energy range, and these parameters have been explicitly matched between GTR2 and GTR4 (${SAD}_{vir}^{X}=221.5 {\mathrm{cm}}$ and ${SAD}_{vir}^{Y}=184.0 {\mathrm{cm}}$ for both gantries) before. Second, for these two gantries, the beam meterset calibrations in beam models were scaled strictly according to the raw measurements of absolute dosimetry, hence there were no computation-measurement absolute dosimetry discrepancies introduced during modeling (the relative differences maintain 0.00% throughout 33 nominal energies at selected depths).

The built-in dose calculation algorithms in RayStation include Pencil Beam (PB) and Monte Carlo (MC) dose engine. Although PB algorithm provides faster computation, it leads to inaccurate dose in some reported cases [9, 10], especially when range shifters are used for small or shallow targets [11]. The manual [12] also strongly recommended using the MC algorithm for final dose computations. Therefore, all involved dose calculations, including computed dosimetric characteristics and patient-specific QA plans, were performed using MC algorithm in the present work.

### Integrated depth dose distribution

In both gantries, IBA Blue Phantom^2^ three-dimensional water phantom and Stingray (IBA Dosimetry, Schwarzenbruck, Germany), a plane-parallel ionization chamber with a diameter of 120.0 mm and an electrode spacing of 1.0 mm, was used to measure the actual IDD curves for 33 selected nominal energy levels (in the range of 70.0 to 225.0 MeV with a step of 5 MeV, as well as the maximum energy 226.09 MeV). The diameter of Stingray is large enough to intercept all beam protons and secondary products [13, 14]. The measurements started at the water surface and included the zero-dose level after the Bragg peaks. The raw measurements of IDD curve for each nominal energy were fitted in RayPhysics using the linear combinations of pre-calculated mono-energetic pristine Bragg peaks during modeling, and their superposition weights referred to the Effective Energy Spectrum for the particular nominal energy.

For visual and quantitative comparison, the computed IDD curves at 33 original nominal energy levels were calculated via MC dose engine. Prior to analysis, the computed IDD curves resolution was spline interpolated from 1.0 to 0.1 mm as the raw measurements, and all curves were renormalized to unity for comparison. With our MATLAB-based (MathWorks Inc, MA, USA) in-house tool, the γ analysis [15, 16] with 1 mm/1% criteria was used to evaluate the intra-gantry agreement between (a) each specific gantry’s measured and computed IDD curves, as well as the cross-gantry agreement between (b) two gantries’ measured IDD curves, (c) two gantries’ computed IDD curves.

### Beam spots

In both gantries, the gantry angle was set as 90°. Five different planes along the beam axis (± 15 cm, ± 30 cm, and the isocenter 0 cm) covering the range of therapeutic interest were selected to measure the planar dose distributions of the 5-spot test pattern [17] at the above-mentioned 33 nominal energy levels. The measurements were performed with LynxPT (IBA Dosimetry, Schwarzenbruck, Germany), a scintillator-based detector with an active detection area of 300 mm × 300 mm and a high effective spatial resolution of 0.5 mm. The detector signal was collected by the CCD with a dimension of 1024 × 1024 and was digitalized at a 10-bit depth. A python-based in-house tool was used to extract and calculate the equivalent average planar dose distribution of the five spots from each 5-spot test pattern, and derive their cross sections separately in X and Y directions.

In RayStation, with a small-angle approximation, the PBS spot profile is most often regarded as a single Gaussian distribution with the mathematical form of 2D cylindrical Gaussian probability density. During modeling, each spot profile was first fitted by a single Gaussian distribution via a least-square fitting, and the standard deviation could be extracted from each fitted Gaussian curve. According to Fermi-Eyges (FE) transport theory [18], considered the free drift condition, the extracted standard deviations at five measured planes were used to fit the FE equation for each nominal energy and obtained the beam optical parameters (i.e., the spatial-angular distribution moments, consist of the angular variance, the spatial-angular covariance, and the spatial variance) for each nominal energy at the isocenter. Theoretically, with these beam optical parameters, the fitted equation could completely describe the transverse spreading during beam transmission at every position along the beam axis—and for more clinical application, at the entry point of the patient.

Likewise, for 5 original selected planes, the computed spot profiles were calculated at 33 nominal energy levels via MC dose engine to reproduce the corresponding raw measurements. We quantitatively compared the intra-gantry deviations between (a) each specific gantry’s measured and computed profiles, as well as the cross-gantry deviations between (b) two gantries’ measured profiles, (c) two gantries’ computed profiles.

### Model-sharing patient-specific QA

The aim of performing model-sharing patient-specific QA is to evaluate the clinical impact of beam model deviations by forcing the 2 gantries to share the same beam model. A total of 36 patients (12 with nasopharyngeal carcinoma (NPC), 12 with central lung cancer, and 12 with prostate cancer) treated with photon radiotherapy since 2019 were selected retrospectively. The CT scanning slice thickness was 3 mm in the head and neck and 5 mm in the other sites. Each patient’s intensity-modulated proton therapy (IMPT) plan was generated and transferred to the water phantom to create the patient-specific QA plan. The plan optimizations and final dose calculations were performed with both GTR2 and GTR4 beam models. The final dose calculation resolution was 1 mm, and this process was completed by two experienced physicists.

Followed this, in GTR2 and GTR4, the patient-specific QA tasks were performed. A water phantom DigiPhantPT with MatriXX PT [19] (IBA Dosimetry, Schwarzenbruck, Germany) was used for planar dose measurements at 3 selected depths. MatriXX PT has an active area of 24.4 cm × 24.4 cm and a pixel spacing of 7.6 mm. The two-dimensional γ analysis in myQA (IBA Dosimetry, Schwarzenbruck, Germany) was applied to evaluate the QA passing rates, with a 10% low-dose threshold and two criteria (2 mm/3% and 3 mm/3%). The results of those QA plans generated with the GTR2 beam model, then delivered in GTR2 and GTR4, are hereafter denoted as *M2G2* and *M2G4*, respectively. Likewise, the results of those QA plans generated with the GTR4 beam model, then delivered in GTR2 and GTR4, are hereafter denoted as *M4G2* and *M4G4*, respectively. Paired t-test and Pearson correlation analysis were used to statistically compare the corresponding results through SPSS (IBM, USA).

## Results

### Integrated depth dose distribution

**Fig. 1:**
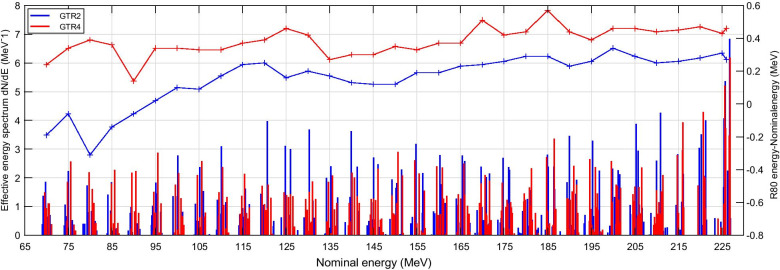
Effective energy spectra and R80/nominal energy difference of GTR2 and GTR4

**Fig. 2:**
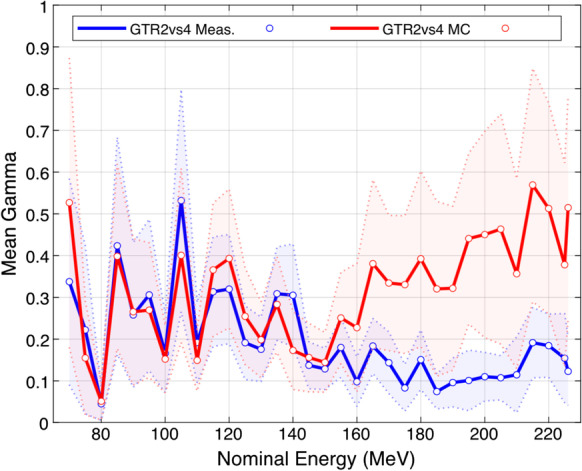
Mean γ indices of each nominal energy in cross-gantry comparisons

**Fig. 3:**
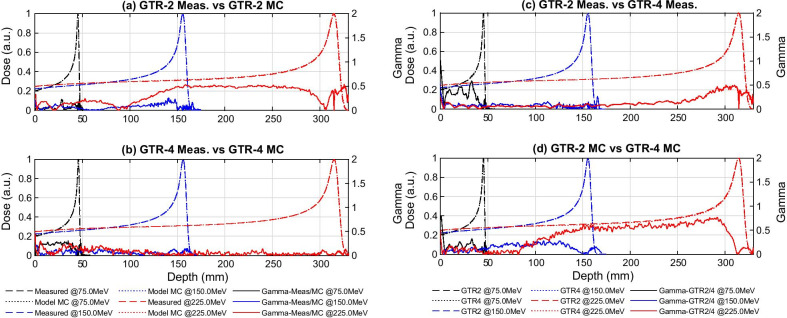
Representative examples of: intra-gantry comparisons between measured and computed IDD curves for each gantry (**a**, **b**), and cross-gantry comparisons of IDD curves between GTR2 and GTR4 (**c**, **d**)

For both gantries, the Effective Energy Spectra together with the R80 Energy, which was defined as the single equivalent energy with the same R80 range as the corresponding energy spectrum [12], are shown in Fig. 1 as the function of the nominal beam energy. The energy of GTR4 tended to be higher than the corresponding nominal energy, and these deviations were larger than those of GTR2. In the intra-gantry comparisons, all the γ analysis between measured and computed IDD curves reached the passing rate of 100.0% with 1 mm/1% criterion. Among them, GTR4 showed a higher computation-measurement agreement. The mean γ index was within the range of 0.03 to 0.17 throughout 33 IDD curves pairs. As for GTR2, its overall computation-measurement agreement was relatively lower, with a mean γ index range of 0.03 to 0.43. In the cross-gantry comparison, for most of the 33 nominal energies the γ passing rates reached 100.0%, only except for 70.0 MeV measured curves comparison (99.1%) as well as 70.0 MeV computed curves comparison (98.8%). Mean γ indices of each nominal energy in cross-gantry comparisons are shown in Fig. 2. It can be seen that the deviations between computed curves were generally larger than the deviations between the measurements, especially in the high-energy region. In other words, errors introduced by the modeling and MC calculation reduced the agreement of the two gantries’ IDD curves. Figure 3a and b show examples of the intra-gantry comparisons at three representative energy levels (75.0 MeV for low, 150.0 MeV for medium, and 225.0 MeV for high energy). Figure 3c and d show corresponding examples of cross-gantry comparisons between two gantries’ measured curves and two gantries’ computed curves.

### Beam spot

**Fig. 4:**
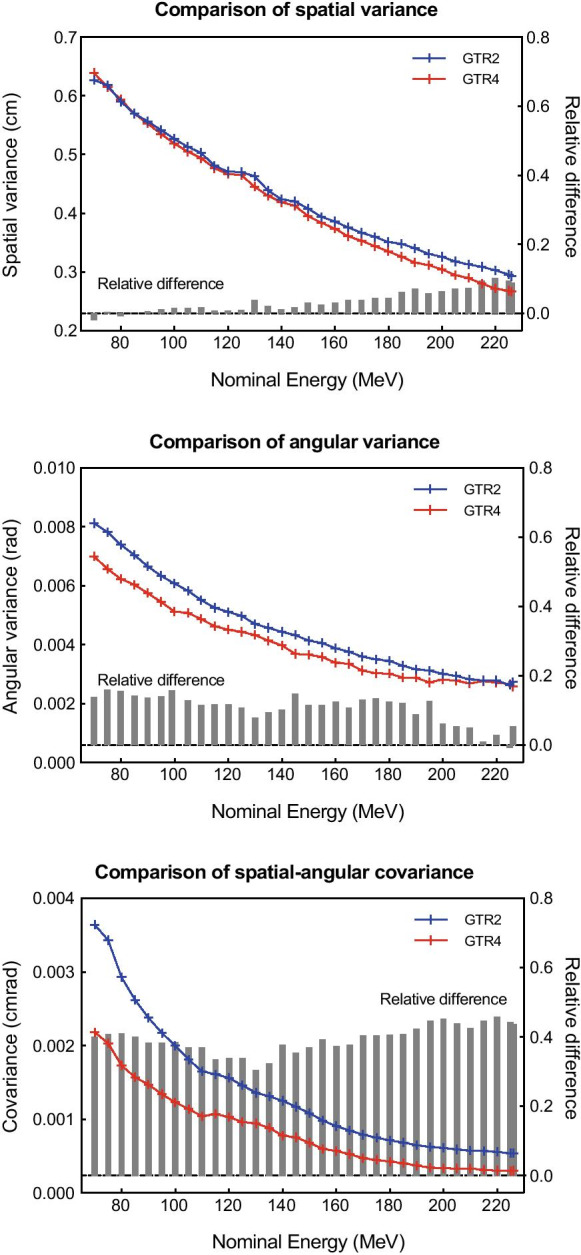
Comparison of the spatial-angular distribution moments for each energy at the isocenter

Three subplots in Fig. 4 separately show the three components of the fitted beam optics at the isocenter, including the spatial variance, the angular variance, the spatial-angular covariance, together with their cross-gantry relative difference between two gantries. The spatial variance absolute difference increased with the nominal energy, while the angular variance and the spatial-angular covariance had an opposite trend. The mean relative difference throughout 33 nominal energies, was 3.64% (− 1.91–10.23%) for the spatial variance, 10.58% (− 0.75–15.98%) for the angular variance, and 39.39% (30.51–45.88%) for the spatial-angular covariance.

**Fig. 5:**
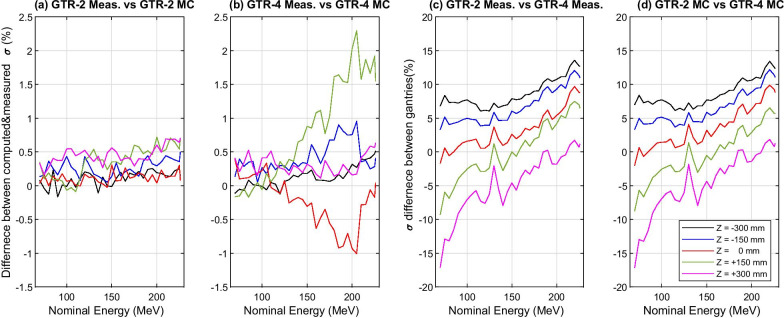
Intra-gantry comparisons between measured and computed spot profile $\sigma$ for each gantry (**a**, **b**). Cross-gantry comparisons of spot profile $\sigma$ between GTR2 and GTR4 (**c**, **d**)

Figure 5a and b show the intra-gantry comparisons between each gantry’s measured and computed beam profiles, by calculating the relative difference of spot profiles’ Gaussian sigmas [20]. For GTR2, the computation-measurement deviations were all within a small range of  − 0.17 to 0.70%, while they were between − 0.93 and 2.30% for GTR4. Even so, these deviations were all small enough to be acceptable. Similarly, the cross-gantry deviations between two gantries’ measured beam profiles, and two gantries’ computed beam profiles, are shown in Fig. 5c and d. Because there were only slight deviations between the measured and calculated profiles, their cross-gantry comparison results were similar. At the isocenter, the cross-gantry deviations were within $\pm 10\%$ throughout 33 nominal energies.

### Model-sharing patient-specific QA

γ analysis results of model-sharing patient-specific QA tasks are listed in Table 1, containing both 2 mm/3% and 3 mm/3% criteria. For all cohorts, the mean passing rate only had slight changes when the beam model or the delivered gantry were changed, and all reached a high level of 95.0%. As for each QA plan, the mean passing rate was not less than 90.0% when using the 2 mm/3% criterion, while that was not less than 95.0% when using the 3 mm/3% criterion, which met the requirements of clinical treatment. Table 2 presents the statistical differences in QA results for each gantry with the usage of two models. Under different criteria, Pearson correlation coefficients showed a moderate-high positive correlation between the QA results using different beam models. In addition, these differences were within the range of ± 0.5%, and paired t-test showed they were not statistically significant (*p* > 0.05).

**Table 1:** Site-distinguished γ results of patient-specific QA

Beam model	Delivered gantry	Malignancy	Criteria	Mean passing rate (%)	Range
Shared GTR2 model	GTR2 (M2G2)	Prostate	2 mm/3%	96.83	90.6–99.4
			3 mm/3%	98.98	96.7–100.0
		Lung	2 mm/3%	97.67	94.3–100.0
			3 mm/3%	99.06	97.4–100.0
		NPC	2 mm/3%	98.36	92.3–100.0
			3 mm/3%	99.69	97.9–100.0
	GTR4 (M2G4)	Prostate	2 mm/3%	97.25	92.8–99.9
			3 mm/3%	99.00	96.7–100.0
		Lung	2 mm/3%	97.91	93.7–100.0
			3 mm/3%	99.19	97.4–100.0
		NPC	2 mm/3%	98.24	92.1–100.0
			3 mm/3%	99.58	98.3–100.0
Shared GTR4 model	GTR2 (M4G2)	Prostate	2 mm/3%	96.74	90.8–99.9
			3 mm/3%	98.91	96.7–100.0
		Lung	2 mm/3%	97.26	92.6–100.0
			3 mm/3%	98.91	96.6–100.0
		NPC	2 mm/3%	98.44	93.0–100.0
			3 mm/3%	99.46	96.3–100.0
	GTR4 (M4G4)	Prostate	2 mm/3%	97.34	93.9–99.7
			3 mm/3%	99.10	96.7–100.0
		Lung	2 mm/3%	97.81	94.1–100.0
			3 mm/3%	98.99	96.7–100.0
		Head	2 mm/3%	98.49	93.1–100.0
			3 mm/3%	99.41	96.4–100.0

**Table 2:** Statistical analysis results of patient-specific QA

Comparison	Malignancy	Criteria	Difference (%)	t/*p*	*r*(*p*)
M2G2 versus M4G2	Prostate	2 mm/3%	0.09	0.442/0.661	0.847(0.00)
		3 mm/3%	0.07	0.907/0.371	0.866(0.00)
	Lung	2 mm/3%	0.41	1.992/0.054	0.829(0.00)
		3 mm/3%	0.15	1.306/0.200	0.754(0.00)
	NPC	2 mm/3%	− 0.09	− 0.229/0.820	0.432(0.00)
		3 mm/3%	0.22	1.865/0.071	0.604(0.00)
M2G4 versus M4G4	Prostate	2 mm/3%	− 0.09	− 0.705/0.486	0.916(0.00)
		3 mm/3%	− 0.10	− 1.399/0.171	0.865(0.00)
	Lung	2 mm/3%	0.10	0.470/0.641	0.788(0.00)
		3 mm/3%	0.21	1.788/0.082	0.732(0.00)
	NPC	2 mm/3%	− 0.25	− 0.809/0.424	0.589(0.00)
		3 mm/3%	0.17	1.367/0.180	0.575(0.00)

## Discussion

In addition to the delivery performance of the system, the accuracy of the TPS model is also a critical part of the therapy. In this paper, we provide a general reference t o the beam-matching application from the model perspective. This work systemically investigated the ability of independently-tuned room-specific beam models of beam-matched gantries to reproduce the agreement between gantries’ performance when considering the errors introduced during modeling, through both intra-gantry and cross-gantry quantitative comparisons, as well as model-sharing patient-specific QA.

Fitting and tuning for beam model parameters, as well as dose calculation, were the main stages that could introduce discrepancies between finally presented computed dosimetric characteristics and the measured ones (i.e., the actual beam properties). Among them, the computation of model-based dosimetric characteristics via MC algorithm, as a means of representing the model fitting results, would cause a slight impact. The computed results could only match the imported measured data to the greatest extent, which mainly depends on the processing of the raw measurements, fitting approach and the parameter fine-tuning during modeling.

In the analysis of IDD curves, the intra-gantry comparison showed that in contrast to the high reducibility of GTR4, the modeling of GTR2 introduced more significant computation-measurement deviations. As for the cross-gantry comparison, it was found that the agreement between the two raw measurements was good, indicating that the two beam-matched gantries had indeed reached a high IDD agreement. However, the difference between the two models was obviously greater, but still reasonable. In addition, regardless of the agreement comparison between the two raw measurements or between two models, unexpected large IDD deviations occurred at 70 MeV in the low-depth range. This was mainly due to factors such as set-up errors having a significant relative influence on the results when the proton beam at such a low energy level had a smaller range. Besides, it could be noted in the intra-gantry comparison; the GTR2 gamma index remained close to 0.5 from around 150 mm to 250 mm in the plateau region at 225.0 MeV (Fig. 3a), which had a similar trend with the gamma curve of the cross-gantry model-model comparison at 225.0 MeV (Fig. 3d). In fact, in our analysis, this phenomenon was common at medium–high energy levels ranging from 165.0 to 226.09 MeV. It proved that the measurement-model discrepancies introduced in modeling were propagated to the cross-gantry model-model comparisons, which affected the agreement between the two gantries’ models. One issue that needs to be mentioned here is the uncertainty of IDD curve measurement, which could be dominated by the positioning accuracy of the water phantom when performing measurement for two gantries. It was reported in the existing literature [13] that the uncertainty is approximately ± 0.3 mm, which could be regarded as the general cross-gantry repeatability. And according to our experience, the uncertainty of intra-gantry repeated measurement could be reduced to ± 0.2 mm. Although the gamma analysis with 1 mm/1% criterion we used might be robust to such distance deviations, it is necessary to introduce a cross-gantry agreement evaluation method that could decouple the comparison of IDD range and curve shape in the future study.

When we studied the beam spot profiles, our approach of using the 5-spot test pattern in each measurement plane to obtain the equivalent mean spot dose distribution, improved the robustness and repeatability for the establishment of symmetric Gaussian beam model, which was demonstrated by previous commissioning tasks [7]. Contrary to the previous analysis of IDD curves, it was found in beam profiles comparison that the discrepancy introduced in GTR4 modeling was significantly greater than that of GTR2. But even if these errors were taken into account, it did not deteriorate the model-model agreement too much compared to the original measurement-measurement agreement. In addition, due to the spot’s evolution along the beam axis, the isocenter was generally not the position where $\sigma s$ of the two gantries differed the most. It was widely used in the clinical stu dies that, the PBS beam spot $\sigma \mathrm{s}$ and symmetries were considered only at the isocenter. This might not be advisable because such consideration actually ignored the beam transmission transverse spread along the beam axis, which could be fully de scribed by the complete beam optics.

The model-sharing patient-specific QA tasks were meaningful supplements to our study, and had the potential to correlate those theoretical parameters with clinical treatment. They checked the agreement between the actual dose delivered results and the TPS dose calculation results. γ analysis showed that the mean passing rate only had slight changes when the beam model or the delivered gantry were changed. Statistical analysis indicated the QA results for a particular gantry with different models were moderate-high correlated, and those slight model-model passing rate differences for each QA plan were not statistically significant, only excepted an outlier *r* = 0.432 when calculated 2 mm/3% QA results’ Pearson correlation coefficients for NPC cases between M2G2 and M4G2. This outlier occurred when the rigorous criterion of 2 mm/3% was used, but when using the less rigorous 3 mm/3% criterion, which was widely used in our clinical practice, there was no exception at all. Although it did not seem to influence clinical treatment, from another perspective, this showed that more detailed differences could only be observed in certain conditions, and need to be further evaluated with more rigorous criteria. In brief, for a particular gantry, the beam model usage for treatment plan generation had no significant effect on the actual delivering performance.

However, the experiments designed in this part had a limitation, that was, we performed planar dose v erification at several selected depths. The sensitivity and reliability of γ analysis had been questioned in previous studies on photon or proton-based plans [21]. In particular, Nelms et al. [22] pointed out that there was a lack of correlati on between conventional planar gamma passing rate and the clinically relevant, anatomy‐based dose errors. And the lack of spatial information in planar comparison was insufficient to analyze the specific causes of measured dose differences [23]. Further studies which are based on proton three-dimensional dose measurement techniques and more rigorous verification criteria are essential.

In the future works, we also plan to artificially increase the measurement-computed (measurement-model) discrepancies, in order to quantitatively explore to what extent, those discrepancies will make the deterioration of the original high agreement between beam-matched gantries not negligible during modeling. Furthermore, at this time, which model (or even have to build and fine-tune a new one) should be selected as the shared model for beam-matched gantries might be considered more carefully.

## Conclusion

The modeling process introduced errors to a certain extent indeed, which made the model-based reproduced results deviate from the raw measurements. Among them, the deviation introduced to the IDD curves was generally larger than that to the beam spots during modeling. From the beam model perspective, the introduced deviations deteriorated the high agreement of the dosimetric characteristics originally shown between two beam-matched gantries, but the cross-gantry discrepancy was still within the clinically acceptable tolerance. In model-sharing patient-specific QA, for the particular gantry, the beam model usage for IMPT QA plan generation had no significant effect on the actual delivering performance. It was preliminarily recognized that among beam-matched gantries, the independently-tuned room-specific beam model from any gantry is reasonable to be chosen as the shared beam model without affecting the treatment efficacy.

## Data Availability

Not applicable.

## Abbreviations

PBS: Pencil beam scanning
QA: Quality assurance
TR: Treatment room
GTR: Gantry
IDD: Integral depth dose
MC: Monte Carlo
ESS: Energy selection system
NPC: Nasopharyngeal carcinoma
IMPT: Intensity-modulated proton therapy
